# An efficient and adaptive test of auditory mental imagery

**DOI:** 10.1007/s00426-020-01322-3

**Published:** 2020-04-30

**Authors:** Rebecca W. Gelding, Peter M. C. Harrison, Sebastian Silas, Blake W. Johnson, William F. Thompson, Daniel Müllensiefen

**Affiliations:** 1 Department of Cognitive Science, Macquarie University, Sydney, Australia; 2 Department of Psychology, Macquarie University, Sydney, Australia; 3 School of Electronic Engineering and Computer Science, Queen Mary, University Of London, London, UK; 4 Department of Psychology, Goldsmiths, University of London, London, UK

## Abstract

The ability to silently hear music in the mind has been argued to be fundamental to musicality. Objective measurements of this subjective imagery experience are needed if this link between imagery ability and musicality is to be investigated. However, previous tests of musical imagery either rely on self-report, rely on melodic memory, or do not cater in range of abilities. The Pitch Imagery Arrow Task (PIAT) was designed to address these shortcomings; however, it is impractically long. In this paper, we shorten the PIAT using adaptive testing and automatic item generation. We interrogate the cognitive processes underlying the PIAT through item response modelling. The result is an efficient online test of auditory mental imagery ability (adaptive Pitch Imagery Arrow Task: aPIAT) that takes 8 min to complete, is adaptive to participant’s individual ability, and so can be used to test participants with a range of musical backgrounds. Performance on the aPIAT showed positive moderate-to-strong correlations with measures of non-musical and musical working memory, self-reported musical training, and general musical sophistication. Ability on the task was best predicted by the ability to maintain and manipulate tones in mental imagery, as well as to resist perceptual biases that can lead to incorrect responses. As such, the aPIAT is the ideal tool in which to investigate the relationship between pitch imagery ability and musicality.

## Introduction

Historically mental imagery has been understood as the representation in the mind of a sensory experience in the absence of sensory input (Kosslyn, 1980). However, more recent theories of embodied cognition suggest that such representations are not limited to the mind only, but are distributed throughout or influenced by the body (Shapiro, 2011). Although ancient philosophers such as Aristotle believed that imagination was central to thought itself (MacKisack et al., 2016), it was not until the 1970s that modern research began to explore the phenomenon of visual imagery (Kosslyn, 1973; Shepard & Metzler, 1971). Visual images can be subjected to a number of operations such as inspection, zooming, rotation, and transformation (Thagard, 2005). However, only in the 1990s was the first volume written on the study of imagery in the auditory modality (Reisberg, 1992).

Musical imagery is often considered a subset of auditory imagery and has been described as the silent mental replaying of music in one’s own mind (Halpern, 2003). However, especially for musicians, musical imagery can involve more than just the auditory modality, with individuals developing multimodal representations of music notation and feeling the body movements implied by the music (Clark, Williamon, & Aksentijevic, 2012). The ability to hear music internally has been argued to be fundamental to musical expertise (Gordon, 1989b; Seashore, 1919), and hence, the earliest application of the study of musical imagery was limited to music education, teaching young musicians to imagine a desired sound and co-ordinate their movement to enable that sound to occur (Goldsworthy, 2010). More recent research has supported this association between imagery and musical skill, showing that musical imagery supports effective ensemble playing (Keller, 2012; Keller & Appel, 2010). Other research has explored the potential benefits of auditory imagery for movement disorders such as Parkinson’s disease and stroke (Lee, Seok, Kim, Park, & Kim, 2018; Schaefer, 2017), memory disorders such as dementia (Halpern, Golden, Magdalinou, Witoonpanich, & Warren, 2015), and the control of auditory hallucinations in clinical and non-clinical populations (Kumar et al., 2014; Linden et al., 2011; Shinosaki et al., 2003). Considering such wide-ranging implications of auditory imagery, efficient and reliable tests of auditory imagery ability are urgently needed.

Development of such tests may also have theoretical implications. Edwin Gordon defined “audiation” as “the hearing of music in one’s mind when the sound is not physically present” (Gordon, 1985, p. 34). The definition then is synonymous with “musical imagery” (Zatorre, Halpern, & Bouffard, 2010), yet to Gordon, audiation was a broader concept involving seven subtypes, that encompassed the processes involved in understanding music that has just been heard, recalling music, composing, as well as performing (Gordon, 1989b). Gordon’s fourth subtype of audiation, namely “recalling familiar music silently”, is, therefore, most relevant to the present study (Gordon, 1985). Gordon theorized that audiation is the central mental facility that represents musical aptitude, and hence designed tests to measure music audiation for all ages of development from pre-schoolers to adults (Gordon, 1989a). Today, these tests continue to be used by music researchers (Burgoyne, Harris, & Hambrick, 2019; Puschmann, 2013), though most recently some have argued that the norms for children and different age groups have not been updated for 3–4 decades and may no longer be valid (Ireland, Parker, Foster, & Penhune, 2018). However, Gordon’s (1985) audiation theory is often overlooked in the current musical imagery literature. The audiation tests that were developed consist of same-different melodic discrimination tests, which have been shown to involve a range of cognitive processes (Harrison, Musil, & Müllensiefen, 2016), and, therefore, are not specific enough to address individual differences. Hence, the development of a more efficient and specific test of auditory imagery may be used to address the theoretical question of whether audiation, specifically the subtype involving auditory imagery, is a main predictor of musical aptitude.

Numerous studies have examined musical imagery abilities, with many investigations focused on their neural correlates (Cebrian & Janata, 2010; Halpern, 1992; Herholz, Halpern, & Zatorre, 2012; Herholz, Lappe, Knief, & Pantev, 2008; Leaver, Van Lare, Zielinski, Halpern, & Rauschecker, 2009; Zatorre & Halpern, 2005; Zatorre et al., 2010; Zatorre, Halpern, Perry, Meyer, & Evans, 1996). However, most studies of musical imagery have explored passive musical imagery, using paradigms requiring continuation of familiar melodies in silence (Herholz et al., 2008; Weir, Williamson, & Müllensiefen, 2015), or comparisons of pitches from lyrics of familiar songs (Aleman, Nieuwenstein, Böcker, & de Haan, 2000; Halpern, 1992). Active musical imagery, which requires manipulation and control over the imagined content, has received less attention (Halpern, 2012; Zatorre et al., 2010). Across both forms, several limitations in the study of musical imagery remain. These include lack of objective measures of performance (Kraemer, Macrae, Green, & Kelley, 2005); and inflexibility—tasks that are too easy for musicians (Janata & Paroo, 2006) and too hard for non-musicians (Zatorre et al., 2010). Other tests have used musical notation to explore musical imagery in musical experts; however, these types of tests are not readily transferable to the general population (Wolf, Kopiez, & Platz, 2018). Given pitch and rhythm are the two primary dimensions of music (Krumhansl, 2000), and imagery performance in these domains has been found to be dissociable, with temporal accuracy often worse than pitch accuracy (Janata & Paroo, 2006; Weir et al., 2015), isolating these two dimensions should be useful for understanding individual differences in musical imagery. The Pitch Imagery Arrow Task (PIAT) was designed to address the former of these dimensions (Gelding, Thompson, & Johnson, 2015); through controlling for other musical features such as rhythm, timbre, and harmony, this task provides a measure of pitch imagery ability.

The PIAT has several advantages over existing protocols for evaluating imagery. Specifically, the task (1) requires a behavioural response to objectively measure accuracy and response times of imagery performance; (2) is extremely difficult to successfully perform using cognitive strategies other than pitch imagery; (3) employs novel rather than familiar sequences of pitches that cannot be anticipated in advance; (4) employs a range of difficulties implemented in a staircase design, such that it can test imagery in participants with a wide range of musical experience. However, one of the main limitations is the time taken to complete the task (approx. 1 h). With 90 trials, the task is time-consuming and experienced as tedious by many participants. Whilst some modified versions of the PIAT have been used (Colley, Keller, & Halpern, 2018; Greenspon & Pfordresher, 2019), they have also been non-adaptive to individual ability.

One way to optimize tests of individual differences, making them more time-efficient and reliable, is through modern psychometric techniques such as item response theory (IRT) and computerized adaptive testing (CAT) (Harrison, Collins, & Müllensiefen, 2017). The main prerequisite for a PIAT version using IRT and CAT is a psychometric model that predicts the difficulty of PIAT items. The aim of the present studies was to construct and validate such a model. First, an exploratory study using the original PIAT tested 115 participants to determine the key variables that contribute to item difficulty. A cognitive model of the processes used to complete a PIAT trial was then developed on the basis of these exploratory results. Subsequently, a calibration study was conducted that systematically tested a large bank of pre-generated items and determined parameters of an explanatory IRT model. This final model serves to construct a CAT version of the PIAT, the new adaptive PIAT (aPIAT) which is both shorter and more efficient. Several studies have shown a link between working memory ability and imagery vividness (Baddeley & Andrade, 2000; Cebrian & Janata, 2010), and an overlap in brain regions responsible for short-term/working memory processes and effortful auditory imagery processes (For review, see Schaefer, 2017). Given that manipulation of auditory images relies heavily on working memory representations (Keller, 2012), and the aPIAT involves manipulation of pitch images, in Study 3, the test–retest reliability and validity of the aPIAT are assessed against a range of musical and non-musical working memory tasks.

## Study 1: exploratory phase

The aim of the first study was to identify features of musical structure and aspects of trial design that contribute to item difficulty on the original PIAT and, hence, to generate an initial psychometric model of task performance on the PIAT.

### Materials and methods

#### Participants

A total of 115 participants completed this study over three recruitment stages. Initial 40 participants (22 females) were recruited for the original PIAT study (Gelding et al., 2015). Additional 24 participants (15 females) completed an identical task as outlined in Gelding et al. (2015), to qualify for a different study. All of these participants (*n* = 64) completed the original version of the PIAT along with two control conditions—perception and mental arithmetic. Perception trials were identical to Imagery trials (described below), but with no arrows presented in silence. Hence, participants matched the audible probe to the last note just heard. Mental arithmetic trials required simple addition and subtraction of ongoing sums as guided by visual presentation of up/down arrows and digits. The remaining 51 participants (35 females) completed the PIAT with only imagery trials included (that is, no mental arithmetic or perception control conditions). This latter group also completed a rhythm imagery task during the experimental session either before or after the PIAT.

#### Materials

##### Pitch Imagery Arrow Task (PIAT)

An individual trial on the PIAT begins with an ascending major scale to provide a tonal context. A start note (either tonic or dominant of scale) is then presented simultaneously with the visual presentation of a dot on the screen. A variable number of up/down arrows are next displayed in random order, with each arrow accompanied by a corresponding pitch that moves up/down the scale in stepwise motion. Pitch changes always match the direction indicated by the arrows. These stimuli are followed by a continuation phase consisting of a number of silent arrows, in which participants are required to imagine the corresponding stepwise changes in pitch. Immediately after the sequence of silent arrows, a pre-probe screen appears, to give participants time to consolidate their current pitch image and prepare to hear the probe. One second later, an audible probe pitch is sounded. Participants are then required to indicate whether the probe matches the final imagined tone. When the probe is incorrect, it is always within the same key signature, so that it is not obviously wrong, and a maximum of 2 steps away from correct answer. A staircase design was used in which all participants began on the easiest difficulty and progressed to increased complexity with accurate responses (2 correct answers or 90% correct on a given stage of the task). See Gelding et al. (2015) for more details of the staircase design.

##### Psychometric questionnaires

As well as completing the PIAT, participants also completed two questionnaires, one to measure musical background and the other to measure auditory imagery vividness and control. First, participants in the first two recruitment stages (*n* = 64) completed a generic musical background survey, from which their years of active musical engagement was calculated. This was then used to calculate a Musical Experience Index (MEI) based on the percentage of life years spent actively engaged in music (i.e., years of musical engagement/age). Participants from the third recruitment stage (*n* = 51) completed only the Goldsmith’s Musical Sophistication Index (Gold-MSI; Müllensiefen, Gingras, Musil, & Stewart, 2014) to obtain a comprehensive profile of their musical skills and experiences. The musical training subscale of the Gold-MSI is of particular importance for the current study given the posited link between the ability to imagine music and the amount of formal musical training received (Aleman et al., 2000). Participants in this third recruitment cohort showed a good spread of musical training background with scale scores ranging from 10 to 44 (mean = 26.5, median = 27, SD = 10.46), which is similar to the distribution of musical training in the general population (median = 27 in Müllensiefen et al., 2014). To equate the two different measures of musical training, an MEI was calculated for the third recruitment cohort by taking their response to the question of years of musical training and dividing by their age. However, the Gold-MSI requires participants to tick a box for the years of musical training, and the category for the longest period of musical training is “10 + years”. Given the minimum age of participants was 18 years, this means that the maximum MEI approximated for the third recruitment cohort was 10/18 = 0.55. This was the case for 12 out of the 51 participants.

Second, all participants completed the Bucknell Auditory Imagery Scale (BAIS; Halpern, 2015). This 7-point Likert scale includes two subscales, for vividness (BAIS-V) and control (BAIS-C), both of which have 14 items each. Participants in this study showed a good range of vividness from 2.85 to 7 (mean = 5.025, median = 4.929, SD = 0.960) and a range of control scores from 3 to 7 (mean = 5.202, median = 5.286, SD = 0.964), which is similar to the distribution of Halpern (2015) who found that both BAIS-V and BAIS-C had mean scores of 5.1 and SD of 0.9.

#### Procedure

Presentation® software (Version 18.0, Neurobehavioral Systems, Inc., Berkeley, CA) was used to control the experiment and to record responses. Acoustic stimuli were generated from the 'Piano' instrument sound by Finale 2012 software (Makemusic Inc; Eden Prairie, MN) and exported as.wav files for use in Presentation^®^.

Upon being seated in front of the computer with headphones, participants were given a sound check, whereby they could manually adjust the volume of the tones to a suitable level. They were then introduced to the task. Participants were informed that no movement or humming was allowed, to assist them with the task, but they should “as vividly as possible, imagine the tones and keep their bodies still”. An opportunity for questions was given prior to the start of the task.

The task has a fast exit in which participants who failed to successfully progress through Level 1 of the Imagery Trials on more than 3 attempts (that is, got more than 18 incorrect responses for Level 1 Imagery Trials) were excused from further trials. Fourteen participants were triaged in this way, having completed a range between 41 and 77 trials at their point of exit. These participants were deemed to have found the task too difficult or failed to understand how to complete it. At each point of failing Level 1, the participants were given the opportunity to ask questions and the requirements of the task were reiterated verbally.

Upon completion, participants were asked verbally to rate how vividly or clearly they formed the musical images during the task (1—not at all vivid; 5—very vivid). They were also asked: “What strategies did you use to complete the musical imagery task?” Verbal responses were recorded by the experimenter. Participants then completed the BAIS and musical experience or Gold-MSI questionnaires (as per Materials section).

#### Ethics

All participants provided written consent and all procedures were approved by the Macquarie University Human Research Ethics Committee.

### Results

In a first step, correct responses of each participant were summed to characterize each individual’s performance on the PIAT. Summed scores ranged from 41.5 to 99% correct responses with a mean of 75.2% (SD = 11.7%) and a median of 75.9% (first quartile at 70% and third quartile at 82.2%). Table 1 shows the correlations between PIAT scores and demographic as well as musical background variables. There were no significant correlations between performance on the PIAT and gender or age (*p* values ≥ 0.62). In contrast, PIAT scores correlated substantially and significantly [all *p* values < 0.005 after correcting for multiple comparisons using Holm’s (1979) procedure] with all indicators of musical background.

**Table 1:** Correlations with performance accuracy

	Age	Gender	MEI	Musical Training Subscale (Gold-MSI)	BAIS-V	BAIS-C
*N*	115	115	115	51	115	115
Performance accuracy [95% CI]	− 0.043 [− 0.224, 0.141]	0.045 [− 0.139, 0.226]	0.534*** [0.389, 0.653]	0.498** [0.258, 0.680]	0.324** [0.150, 0.479]	0.386*** [0.218, 0.531]

Significance is denoted as **p < 0.01, ***p < 0.001

In particular, the correlation with the aggregated number of years of active musical training/engagement (MEI) of *r* = 0.53 (*p* < 0.001) and the correlation with the Musical Training subscale of the Gold-MSI of *r* = 0.50 (*p* < 0.01) reflect the predicted association between musical training and musical imagery ability (Aleman et al., 2000).

In a second step, data at the level of individual trials were analysed using the packages *lme4* (De Boeck et al., 2011), *AICcmodavg* (Mazerolle, 2017), and *psyphy* (Knoblauch, 2014) in the statistical computing environment R (R Core Team, 2014). These models took the form of mixed-effects logistic regressions, where the outcome variable was trial success (0 or 1). Categorical variables were dummy-coded. We used a model selection strategy based on minimising the corrected Akaike Information Criterion (AICc) as described in Long (2012); the resulting model parameters are listed in Table 2. (See Appendix 1 for the full description of all parameters used. Parameters were identified retrospectively as features of the task that could be manipulated to impact item difficulty).

**Table 2:** Generalized mixed-effects regression model for performance accuracy with 95% confidence intervals

Predictor	Definition	*B*	SE	*z*	*p*
(Intercept)		1.401 [0.999, 1.80]	0.205	6.822	< 0.001***
Level	Number of imagined tones (i.e., silent arrows) per trial	− 0.357 [− 0.463, − 0.251]	0.054	− 6.574	< 0.001***
Probability_Probe	Probability of the probe, given the total number of arrows presented in the trial	2.926 [2.344, 3.508]	0.297	9.848	< 0.001***
ProbeNote_ is_StartNote	A binary variable indicating whether the probe note was identical to the start note of the sequence	− 0.680 [− 0.927, − 0.433]	0.126	− 5.407	< 0.001***
Stage 2	Factor describing trials where start note is tonic, and number arrows in set-up sequence is 3–5	− 0.070 [− 0.286, 0.146]	0.110	− 0.631	0.528
Stage 3	Factor describing trials where start note is dominant, and number arrows in set-up sequence is 3–6	0.119 [− 0.130, 0.368]	0.127	0.934	0.350
Stage 4	Factor describing trials where start note is tonic or dominant, and number arrows in set-up sequence is 3–6. Stage 4 trials only reached when participants successfully completed Level 5–Stage 3	0.772 [0.186, 1.358]	0.299	2.583	0.010**

Statistical significance is denoted as **p < 0.01, ***p < 0.001

The best model (see Table 2) included random effects for participants and items, as well as 6 fixed effects for (1) Level (i.e., the number of silent arrows), (2) the probability of the probe, given the total number of arrows presented in the trial, (3) a binary variable indicating whether the probe note was identical to the start note of the audio–visual sequence, and 3 factors for the different Stages of the trial, that represent variability in start notes and number of heard tones/arrows in the set-up component of a trial (for more detail on the Level/Stage structure of the staircase design, see Gelding et al., 2015). The lower asymptote (guessing level) and the upper asymptote (ceiling level) of the model were optimized given these fixed and random effects and optimal values were identified at 0.3 (floor) and 0.95 (ceiling). Using tenfold cross-validation, the classification accuracy of the final model was 64.9% without random effects (i.e., not using model-based ability estimated from the same participants) and 71.6% with random effects (i.e., using model-based ability estimated from the same participants).

### Discussion

The results of the exploratory study show that there are considerable individual differences between participants on the PIAT and that task performance is significantly correlated with musical training and self-reported ability to imagine auditory material. In addition, data modelling at the individual trial level showed that meaningful factors that affect task difficulty can be identified. Results of the model evaluation demonstrate that these factors (i.e., fixed effects) explain a sizeable proportion of model accuracy (64.9%). Including personal information (i.e., random effects of participant ability) further increases model accuracy to 71.6%. The sizable contributions of individual differences on the task suggest that it is especially suitable for computerized adaptive testing.

The largest predictor of item difficulty was the number of tones that the participant had to imagine: more tones led to higher difficulty. The second largest predictor was the proportion of other items in the item bank that shared the same probe tone (Probability_Probe): less frequent probe tones led to higher difficulty. The probe note was calculated in terms of steps away from the start note, and given the various possible arrow combinations, there was higher probability of the probe note being closer to the start note than at the extremes of the tonal pattern. Repeated exposure to the tones surrounding the start note may have made more frequent probe tones easier or may have biased the participant to expect more frequent probe tones. In addition, we found fewer correct responses for trials where the probe tone was identical to the first tone of the sequence, which suggests a perceptual bias when the start note is used as the probe. That is, for incorrect probes when the probe was the start note, participants were more likely to select it as correct and, therefore, make an error. This confound of task difficulty can be removed by ensuring that trials do not have the probe as the start note. Finally, simpler trial stages (fixed start note and less variability in number of heard tones/silent arrows in set-up) proved to be easier for participants.

Taken together, the results of the exploratory study suggest that it is a well-suited task for constructing an effective test of pitch imagery ability based on a rigorous item response model. Results of the exploratory study also help to construct a hypothetical cognitive model of task performance on the PIAT, which serves as the basis for the subsequent calibration study.

### Cognitive model

**Fig. 1:**
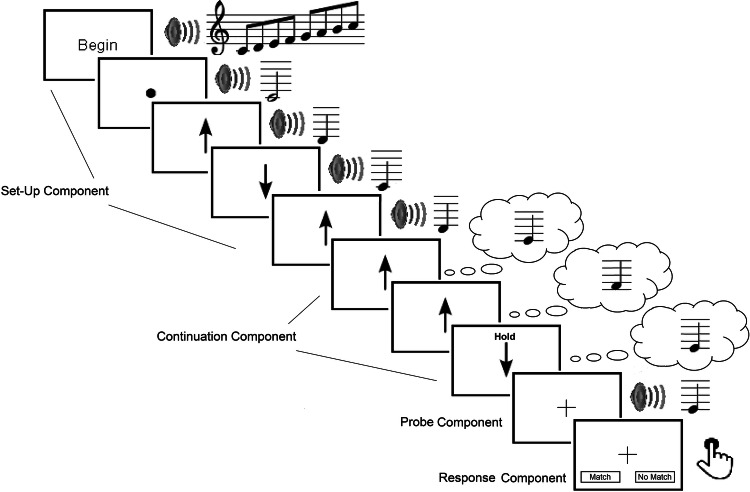
Schematic of the updated PIAT trial. In the schematic, the imagined note matches the actual sounding probe tone. Hence, this example represents a correct probe trial, and the participant should respond with “Match”. The Begin display/ascending scale, start note/black dot, and hold arrow were displayed for 2 s, while all other arrows (with and without sounded notes) were displayed for 1 s

To simplify a PIAT trial, improvements were made to probe and response components of the trial. The original PIAT involved a pre-probe screen to alert participants for the need to maintain the current image and prepare them to hear the probe, which occurred 1 s later (Gelding et al., 2015). In the updated PIAT trial, the pre-probe screen was removed, and instead, the final silent arrow included the word “hold” on it and was displayed for 2 s instead of 1 s. A white cross appears on the screen when the probe is sounded (see Fig. 1). The participants then answered the question “Did the final tone match the note you were imagining?”, with two buttons at the bottom of the screen (“Match” or “No Match”) to choose from.

**Fig. 2:**
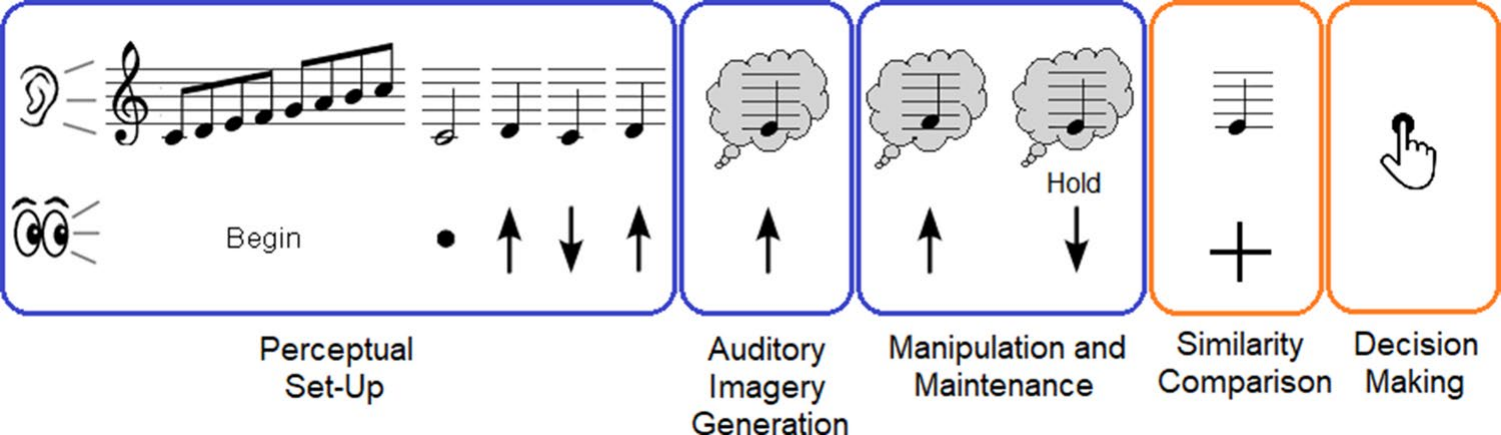
Schematic of the cognitive process model for the PIAT. Blue outlines represent processes of the model (Perceptual Set-Up, Auditory Imagery Generation, Manipulation, and Maintenance) that are the same for all trials, regardless of the probe accuracy. Orange outlines represent the processes of the model (Similarity Comparison and Decision-Making) that vary depending if the probe is correct or incorrect

Using the participant’s descriptions of the strategies used to do the task, as well as common sense in stepping through the thought process of completing the task, a cognitive process model was developed. The purpose of the cognitive process model was to describe the stages of processing of a PIAT trial, to consider how different variables may be related to item difficulty, and, therefore, inform the future calibration modelling (Harrison et al., 2016). The cognitive process model included the following stages: perceptual set-up, auditory imagery generation, manipulation and maintenance, similarity comparison, and decision-making (see Fig. 2).

Perceptual set-up occurs as the participant activates the tonality template for the trials from the presentation of the initial scale and start note. Next, coordinated audio–visual processing is activated through the arrows and tones being presented together during the set-up component. Generation of the first auditory image occurs when the first silent arrow is presented. Given the uncertainty of when the first silent arrow will occur, expectation for a silent arrow increases once the initial number of heard arrows reaches 3, given that all trials had at least 3 sounded arrows in the set-up component. Subsequent processing of the silent arrows guides the manipulation of the auditory image. When the arrow with “Hold” appears, participants then maintain the last imagined note in working memory. A similarity comparison is made when the probe is heard, with a participant then making the decision whether the probe matches the last note which they were imagining.

Item features that impair the imagery stages of the PIAT cognitive process model should increase item difficulty. For example, if the correct auditory image is not originally generated, then subsequent manipulations would lead to an incorrect response. Hence, if participants fail to complete Level 1 items correctly, this suggests a lack of ability in generating a correct auditory image. Errors can also occur during manipulation, if participants are not paying full attention to the silent arrows (and lose one or more steps), if manipulations are performed incorrectly with more than a single step taken with each arrow, or if their imagery strength diminishes over the trial, leading to an impoverished or incorrect image being maintained during the pre-probe period. These types of errors are more likely at higher levels. In such cases, participants may use the information still available to them to complete the task, some of which may cause biases in responses. For example, memory for important notes from the heard sequence (e.g., the tonic or fifth of the scale presented or indeed any note contained in sequence) may bias participants to respond as “match” if imagery for the last note is not strong enough to compare to the probe, and the probe instead matches an important note from the sequence (Deutsch, 1970, 1972). This bias would increase accuracy for correct probe trials but results in errors for incorrect probe trials. Having several steps in one direction within a trial may also increase item difficulty as the correct probe would be further away from the last note heard (hence, items with a larger distance between last heard note and probe would be more difficult).

Other information available to participants if they lose their imagery may be the approximate direction of the probe relative to the last note heard, which could be tracked through counting arrows. If the direction of the probe relative to the last note heard is consistent with the direction of the arrow count (i.e., if the probe is above last heard note, and arrow count is positive), then incorrect probe trials will be more difficult to detect, leading to increased errors. Conversely, if the direction of the probe relative to the last note heard is inconsistent with the direction of the arrow count (i.e., if probe is above last heard note, and arrow count is negative), then incorrect probe trials would be much easier to detect. Incorrect probe trials should also be more difficult if the probe is 1 step rather than 2 steps away from the true imagined note, as the further away the probe is to the true imagined note, the more obviously wrong it will be. The final information participants may also be using in lieu of accurate imagery representations is implicit probe probability approximations, to decide on the likelihood of a given probe being correct, either based from the last note heard or the start note or the total number of arrows in the trials.

Once the probe is sounded, participants compare their imagined note with the probe and must decide whether it is correct. If the imagined probe matches the sounded probe, then a correct decision is straightforward. If it does not match, participants consider their confidence in their imagined note, and the other information at hand, to determine whether to select “no match” or whether they have made an error in their imagery and should instead respond as “match”. Confidence in a response should be highest when the true imagined note matches the last note heard, or when true imagined note is tonic or dominant. Hence, this cognitive model suggests that any explanatory model of data collected from the PIAT should consider correct probe trials and incorrect probe trials separately, and that there are many variables that can be extracted from a trial that could potentially predict item difficulty. These variables have been listed and described in Appendix 1 and were derived as any features of an item/trial that could be quantified, that may contribute to item difficulty. Whilst confidence was not measured as part of the PIAT response, future studies could explore continuous confidence ratings along with binary “match” and “no match” responses.

## Study 2: calibration phase

As a result of the exploratory phase and the development of the cognitive model, several changes were made to the PIAT and a calibration study was conducted. The aim of the calibration study was to explore how item difficulty relates to the different features of a new set of experimental stimuli (*N* = 3000 items). In this new set, the stimuli systematically vary on predictors identified as important in the exploratory phase. The output of the calibration phase is an improved explanatory model that can form the basis for the adaptive version of the PIAT (aPIAT).

### Materials and methods

#### Participants

243 participants with a mean age of 21.8 years (SD = 3.8) took part in the calibration study. 156 (65%) of participants were female and 81 (33.3%) were male, while 3 indicated their gender as “other” and 3 preferred not to disclose their gender. Ten participants were recruited among first-year undergraduates at Goldsmiths University of London who participated for course credit and 233 were recruited through the online panel of the consumer insights company SoundOut and received a small monetary compensation.

#### Materials

Trials were pre-generated as movies using open-source software Openshot (www.openshot.org) and FFMPEG (www.ffmpeg.org), with piano tones from the *Alicia’s Keys* piano plugin (Native Instruments GmbH) for Audacity (www.audacityteam.org). Stimuli were generated to systematically vary level (number of silent arrows in a trial from 1 to 5), number of heard arrows (3–5), start notes (tonic or dominant), accuracies (correct or incorrect), as well as key signatures (C, C#, D, Eb, and E Major) (Janata & Paroo, 2006). Hence, there were 5 (levels) × 3 (heard arrows) × 2 (start notes) × 2 (accuracies) × 5 (keys) = 300 trial types. There were ten variations of each trial type, from the random generation of arrow combinations, resulting in 3000 stimuli being created. The only constraint was that the probe could not be the start note of the trial, and the range of notes over the trial was bounded by ± 4 steps from the start note. This was increased from ± 3 notes from the exploratory study to increase variability and decrease the probability of a given probe.

The presentation of the stimuli was through a custom-made interface implemented using the *psychTestR* package (Harrison, 2019) and delivered through the in R Shiny interface (https://shiny.rstudio.com/).

In addition, participants completed the Gold-MSI self-report questionnaire on their musical background, skills, and expertise (Müllensiefen et al., 2014). Musical training of participants in this study was lower (mean = 23.37, median = 23) compared with the exploratory study. Gold-MSI musical training scores ranged from 7 to 49 with a standard deviation of 9.78.

#### Procedure

Participants were introduced to the task in several steps, explaining the set-up of the tonal context, the alignment of visible arrows and audible tones on the scale, and, finally, the silent arrows that require imagery of the corresponding tones. Participants were then presented with three practice trials where they were given feedback on their responses and were offered the opportunity to repeat the practice trials as many times as they wished. Subsequently, participants had to respond to 30 trials on the PIAT without any feedback. Items were presented quasi-randomly, with the only constraint being that participants received an equal number of items (*n* = 6) from each of the 5 levels. Finally, participants completed the Gold-MSI self-report questionnaire as well as basic demographic questions and asked to describe the strategy that they used to complete the task by selecting one choice from of a list of options.

#### Ethics

The experiment received ethical approval from the Ethics committee at Goldsmiths, University of London.

### Results

The data analysis aimed to construct an explanatory item response model (De Boeck & Wilson, 2004) using a binary logistic regression model with the response score (correct/incorrect response) as dependent variable and 24 variables as potential predictors reflecting different musical or procedural aspects of the individual trials. In addition, the explanatory item response model also ought to include parameters for the lower and upper asymptote of the logistic function to model floor and ceiling performance on the task (i.e., participants’ ‘guessing’ and ‘inattention’ behaviour). Hence, this explanatory item response model does not translate directly to a simple Rasch model, but can be considered a modified Rasch model or a 4PL item response model (Barton & Lord, 1981) with constant values for the discrimination, guessing, and inattention parameters. The predictor variables were derived from the cognitive process model described in Fig. 2 and a short definition of each predictor variable is given in Appendix 1.

To reduce the number of potential predictor variables, we performed an initial variable selection procedure employing random forest classification (Breiman, 2001) to predict the correctness (0 or 1) of the responses at the individual trial level. Random forests have the advantage that they can handle a large number of predictors and provide an index of the importance of each variable for the classification accuracy of the model. We used several measures of variable importance based on random forest models: (1) the mean decrease in model accuracy upon variable permutation, using Breiman’s (2001) original random forest implementation; (2) the mean decrease in model accuracy upon variable permutation, using the random forest implementation based on conditional inference tests as described by Strobl, Malley, & Tutz (2009); (3) the AUC-based variable importance measure upon variable permutation suggested by Janitza, Strobl, and Boulesteix (2013) which is also derived from the random forest implementation based on conditional inference tests (Strobl et al., 2009).

In accordance with the cognitive model, we allowed predictor variables to have different functions (i.e., coefficients) when modelling trials with a correct probe vs trials with an incorrect probe. To this end, we created two data subsets for correct probe trials (3645 observations) and incorrect probe trials (3645 observations). Considering the overall aim to obtain a compact model of the data suitable as a basis for an adaptive test, we selected the ten most important predictors from each of the three random forest models for each data set. Since the three sets of important variables showed a considerable overlap, the combined sets of the most important predictors contained 12 unique variables for each of the two data sets with correct and incorrect probe trials.

The second variable selection step made use of generalized linear mixed-effects models, which are able to account for individual differences by including a random effect for participants to represent participant ability. For each of the two data sets, we constructed a null model (not including any predictor variables as fixed effects, but only the random intercept effect for participants) and a full model including all predictor variables as fixed effects. The parameters for the lower asymptote (guessing parameter) and upper asymptote (inattention parameter) were optimized for each model separately. In a final step, we performed an exhaustive search through all possible subsets of predictor variables as main effects and subsequently optimized the parameters for the lower and upper asymptote again. The best model (according to the Bayesian Information Criterion, BIC) for correct probe trials contained two predictor variables (ProbabilityProbe_LastHeard and Level) and had a much better fit to the data (BIC = 4492.411) than the null (BIC = 4509.053) and the full model (BIC = 4556.578). The classification accuracy of this model, determined on the same data set, was 70.8%. As per Appendix 1, ProbabilityProbe_LastHeard is defined as the probability of probe based on the distance between last heard note and the probe across the total data (3000 items).

The final model for incorrect probe trials contained two predictor variables (ProbeTrueIm_AbsDiff and Heard_Range). As per Appendix 1, ProbeTrueIm_AbsDiff is defined as the absolute difference between the true imagined final note (that should have been imagined, given the presentation of the arrows) and the probe presented, hence is 0 for correct probe trials, but takes a value of 1 or 2 on incorrect probe trials. Heard_Range is defined as the number of unique tones played during the set-up period, including the start note. This model also had a much better fit to the data (BIC = 4856.6) than the corresponding null model (BIC = 4903.967) and full model (BIC = 4909.979). Its classification accuracy was 66.83%.

In a final step, we combined the predictor variables from both models into a single model specifying an interaction effect of each predictor with the status of the probe (correct/incorrect). Coefficients for all predictors and parameters for the lower and upper asymptote were estimated on the full data set (7290 observations). The final model had a prediction accuracy of 63.8%.

Table 3 provides summaries of all three models (i.e., correct probe trials, incorrect probe trials, and joint model). The model summaries show that some of the predictors assume different functions for correct and incorrect probe trials. ProbeTrueIm_AbsDiff is only meaningfully defined for incorrect probe trials, while Heard_Range has a negative coefficient for incorrect probe trials and a positive coefficient for correct probe trials. For ProbabilityProbe_LastHeard, only the coefficient for correct probe trials is statistically significant. In contrast, level has coefficients of similar magnitude for correct and incorrect probe trials, both of which are statistically significant.

**Table 3:** Generalized linear regression model predicting item difficulty from correct probe trials, incorrect probe trials, and the joint model

Predictor	*β* [95% CI]	SE	*z*	*p*
**Correct probe trials**				
(Intercept)	0.839 [0.535, 1.143]	0.155	5.411	< 0.001***
ProbabilityProbe_LastHeard	1.052 [0.364, 1.740]	0.351	2.999	0.003 **
Level	− 0.116 [− 0.169, − 0.063]	0.027	− 4.332	< 0.001***
**Incorrect probe trials**				
(Intercept)	− 1.633 [− 2.303, − 0.963]	0.342	− 4.770	< 0.001***
ProbeTrueIm_AbsDiff	1.028 [0.763, 1.293]	0.135	7.607	< 0.001***
Heard_Range	− 0.224 [− 0.377, − 0.071]	0.078	− 2.886	0.004 **
**Joint model**				
(Intercept)	− 0.918 [− 1.516, − 0.320]	0.305	− 3.009	0.003 **
Incorrect: ProbabilityProbe_LastHeard	0.228 [− 1.277, 1.733]	0.768	0.296	0.767
Correct: ProbabilityProbe_LastHeard	2.778 [1.602, 3.954]	0.600	4.627	< 0.001***
Incorrect: level	− 0.157 [− 0.282, − 0.032]	0.064	− 2.438	0.015 *
Correct: level	− 0.176 [− 0.266, − 0.086]	0.046	− 3.795	< 0.001***
Incorrect: ProbeTrueIm_AbsDiff	1.151 [0.820, 1.482]	0.169	6.823	< 0.001***
Incorrect: Heard_Range	− 0.553 [− 0.763, − 0.343]	0.107	− 5.164	< 0.001***
Correct: Heard_Range	0.157 [0.014, 0.300]	0.073	2.162	0.031 *

In the joint model, correct indicates Probe_Accuracy = 1; incorrect indicates Probe_Accuracy = 0

*p* values are estimated using Wald tests

The selected predictors and the signs of their coefficients for correct and incorrect probe trials are consistent with the cognitive model. Higher levels (i.e., more imagined tones) led to a lower performance which indicates that longer sequences make it more likely that participants can lose their imagery or imagine notes that are not congruent with the arrows shown. This applies to correct and incorrect probe trials alike. If participants are not able to correctly imagine the sequence of tones, they then must rely on alternative cognitive and perceptual heuristics. These heuristics include the probability of the probe given the number of arrows and the last note of the sequence heard, as well as the most salient traces in auditory working memory, such as notes that were heard during the set-up sequence.

The presence of perceptual bias in responding is evidenced by the significance of predictor ProbabilityProbe_LastHeard in both the model for correct probe trials only and in the correct probe trials within the joint model, but not in incorrect probe trials. This variable is the probability of the probe given the last note heard, calculated from the whole data set of 3000 items. While we do not assume that participants were using statistical learning necessarily to inform their decisions, in practice, the higher probability combinations were those with probe notes closer to the last note heard, but with reasonable distances given the number of silent arrows. That is, with an even number of steps imagined, the correct probe had to be an even number of steps away from the last tone heard, whereas with for odd numbered tones imagined, it was an odd number of steps. Hence, the last note heard and probe combinations that have higher probability were more likely to be selected as a “match” by participants due to the proximity of a prominent note in the trial (i.e., last note heard). When the probes were correct, this means that the bias works in favour of the participants, leading to a significant positive predictor of performance being higher probability of probe; however, when the probes were incorrect, this bias works against the participants, inducing a higher probability of response error.

As predicted in the cognitive model, trials are significantly easier when the probe is two steps away from the true imagined note rather than one step away. This is seen in the variable ProbeTrueIm_AbsDiff, which is statistically significant in the incorrect probe trial only model, and the joint model for incorrect probe trials.

Heard_Range is another variable which suggests the presence of perceptual bias in responding. For the incorrect probe trial only model, this variable significantly and negatively predicts performance. In the joint model again for incorrect probe trials, the coefficient is significant and negative, whereas for correct probe trials in the joint model the coefficient is significant and positive. This suggests that when the Heard_Range is larger (i.e., the difference between the lowest and highest notes of the set-up period is greater), participants are more likely to select the probe as a “match”, leading to correct responses for correct trials, but incorrect responses for incorrect trials.

In a last step, performance on the PIAT was related to participants’ musical background. Performance on the PIAT was measured both by summing the correct responses and by extracting the random effects coefficients from the mixed-effects model, which represent the latent variable of participant ability. Sum scores ranged from 26.7 to 100% correct responses with a mean of 59.5% (SD = 13.5%) and a median of 60% (first quartile at 50% and third quartile at 67.7%). Participants’ random effects ranged from − 1.78 to 3.02 with a mean of 0.057 (SD = 0.87) and a median of − 0.044 (first quartile at − 0.56 and third quartile at 0.56). The correlation between these two indicators of performance was *r* = 0.94. Table 4 shows the correlations between PIAT sum scores and random effects ability scores and demographic as well as musical background variables. There were no significant correlations between performance on the PIAT and gender or age (*p* values ≥ 0.38). In contrast, PIAT scores correlated substantially and significantly (all *p* values < 0.001 after correcting for multiple comparisons using Holm’s (1979) procedure) with self-reported perceptual abilities, emotional musical engagement, and musical training. However, no significant correlations were found with self-reported active engagement, singing abilities, or general sophistication.

**Table 4:** Correlations and 95% confidence intervals with performance accuracy and latent variable participant ability

	Performance accuracy		Participant ability	
Age	− 0.037	[− 0.162, .089]	< .001	[− 0.125, 0.126]
Gender	− 0.042	[− 0.168, .084]	− 0.057	[− 0.182, 0.069]
Active engagement	0.162	[0.037, 0.282]	0.146	[0.021, 0.267]
Emotions	0.296***	[0.177, 0.407]	0.301***	[0.182, 0.411]
Musical training	0.269***	[0.148, 0.382]	0.262***	[0.141, 0.375]
Perceptual abilities	0.333***	[0.216, 0.440]	0.328***	[0.211, 0.436]
Singing abilities	0.142	[0.016, 0.263]	0.125	[− 0.001, .247]
General sophistication	0.151	[0.026, 0.272]	0.124	[− 0.002, 0.246]

Significance is denoted as ***p < 0.001 [corrected for multiple comparisons using Holm’s (1979) procedure]

### Discussion

The calibration study resulted in an explanatory item response model, taking the form of a mixed-effects logistic regression, that explains performance on the PIAT through four aspects of musical structure. As found in the exploratory study, task difficulty increased with the number of imagined arrows (Level), regardless of whether the probe matched the correctly imagined note or not. However, the variables capturing the heard range of notes in the set-up period, and the probability of the probe given the last note heard, differed in their function for trials with correct and incorrect probes, which is indicative of a perceptual bias towards higher probability probe tones and an association of large heard range with the “match” response. Incorrect trials with a probe that was one step away, rather than two steps away, from the correct imagined note also contributed to item difficulty.

This explanatory model, therefore, defines ability on the PIAT as the ability to maintain and manipulate tones in mental imagery as well as to resist perceptual biases that can lead to incorrect responses. In this respect, the model is in line with the recent approaches (Thomas et al., 2018) that combine item response theory and signal detection theory (SDT). In standard SDT, test performance is defined as a measure of participant ability and response bias, with the purpose being to remove response bias, to obtain a more accurate measure of true ability (Thomas et al., 2018). However, our explanatory model incorporates perceptual bias rather than eliminates it, by defining ability on the PIAT as ability to resist perceptual biases and to perform the pitch imagery task correctly. This incorporation of perceptual bias is particularly relevant to music cognition, as going against and playing with perceptual biases and expectation is part of active and passive musical behaviour (Aydogan et al., 2018; Herrmann, Henry, Haegens, & Obleser, 2016).

The model has an acceptable prediction accuracy and is plausible in terms of the suggested cognitive task performance model of the PIAT. In addition, model-based ability estimates along with sum scores from the test correlate significantly with self-reported musical training and perceptual abilities. However, performance on the PIAT is not associated with age nor gender, suggesting that the PIAT represents a fair test with respect to these two variables. The explanatory model was, therefore, adopted for the new computerized adaptive version of the PIAT (aPIAT), which we sought to validate in Study 3.

## Study 3: validation of aPIAT

The main aim of the final study was to validate the new aPIAT against established measures of musical and non-musical working memory (WM). As the processing of items on the aPIAT relies on the general capacity of an individual to hold and manipulate stimuli in memory, we expected moderate correlations with tests of visuo-spatial and digit working memory. In addition to general WM capacity, the processing of aPIAT items also benefits from specific musical knowledge structures, and hence performance on the aPIAT should be correlated with other musical WM tasks. Correlations with musical WM tasks are expected to be stronger than for general or non-musical WM tasks. As per the results of Study 1, we predicted that the aPIAT score would also positively correlate with the amount of musical training and general sophistication individuals self-report on the Gold-MSI, as well as with auditory imagery vividness and control as measured by the BAIS.

The secondary aim of Study 3 was to assess the reliability of the aPIAT. The assessment yields an indication of the test’s measurement error which can then be taken into account in future studies that employ the aPIAT as part of a larger test battery.

Finally, we further explore how manipulating the number of items within the aPIAT impacts upon the test’s validity and reliability. Shortening tests generally reduces validity and reliability (Kruyen, Emons, & Sijtsma, 2013). Hence, it is useful to quantify this effect, so that researchers can balance these reductions in validity and reliability with the practical utility of shorter test lengths.

### Materials and methods

#### Participants

146 participants with a mean age of 26.41 years (SD = 7.73) took part in the validation study. 88 (60.3%) of the participants were female, 56 (38.4%) were male, and 2 (1.3%) did not indicate either gender. 102 participants were recruited from among undergraduate and graduate students as well as older adults living close to Goldsmiths, University of London who participated for course credit or were received a monetary compensation. Forty four were recruited from participant pools at Macquarie University and received either course credit or small monetary reimbursement. Participants were recruited over the age range of 18–50 years with a mix of musical training backgrounds.

#### Materials

##### aPIAT

The aPIAT used in this study was the computerized adaptive test (CAT) version based on the explanatory item response (IRT) joint model as given in Study 2 (Table 3), which was used to generate IRT parameters for the item bank. Given that some participants had reached ceiling levels of performance in the calibration phase, we aimed to increase the range of item difficulty of the test by generating 600 new items in addition to the 3000 items already contained in the item bank. The newly generated items were all Level 6 (i.e., 6 imagined arrows per trial), because (according to the joint IRT model) an increased number of arrows were linked with a reduced proportion of correct responses. The resulting item difficulty parameters incorporated the fixed effects from the joint IRT model as specified in Table 3. As is conventional in IRT, the parameters were scaled, so that a distance of one unit on the difficulty scale corresponded to the standard deviation of participant ability in the sample group. Item selection for consecutive trials was guided by Bayes modal ability estimation, with ability estimates being recalculated after each participant’s response. Each successive item was selected using Urry’s rule (Magis & Raîche, 2012). Final abilities were estimated using weighted likelihood estimation (Warm, 1989) and the outcome measure is a score ranging from − 4 to + 4. The number of test items was set to 25 to limit the overall duration of the test to about 8 min, which includes around 3 min of instructions and training items at the beginning. While longer test lengths generally increase the psychometric properties of the test (i.e., reduction in measurement error and increased reliability), we deliberately aimed for a realistic test duration suitable for individual tests that form part of larger batteries.

##### Non-musical working memory tasks

*Backwards Digit Span (BDS)*: BDS tasks represent a classic measure of WM (Case & Globerson, 1974). The task requires participants to remember a sequence of digits, mentally reverse the sequence, and enter the reversed sequence by clicking the numbers on a keypad. This BDS task was a re-implementation on the BDS used by Vock and Holling (2008) and consisted of 12 trials of increasing difficulty using sequences with 4–7 digits.

*Memory Figural Updating (MUF)*: Visuo-spatial tasks are designed to measure the visuo-spatial scratchpad element of Baddeley’s WM model (Baddeley, 2012). The MUF task is a visuo-spatial task similar to the task used in Salthouse, Babcock, and Shaw (1991) and is also a re-implementation of the test designed by Vock and Holling (2008). Participants were presented a variable number of rectangles where dots could appear in any corner for 1.5 s at a time followed by arrows pointing to other corners of the same rectangles. Participants had to remember the various dot locations and imagine where the dots would move to, based on the arrows shown. Participants responded to each item by clicking the corners of empty rectangles indicating the final position of each dot. The MUF comprized 14 items which increased in difficulty based on the number of mental operations to be completed. The MUF task bears some resemblance to the aPIAT, because participants are instructed to imagine the dot moving to different corners of a rectangle as indicated by a sequence of arrows. However, in contrast to the aPIAT, the MUF is a purely visuo-spatial task with no reference to any musical elements.

*Jack and Jill (JAJ)*: The JAJ measures visuo-spatial WM capacity based on a dual-task paradigm. Participants have to hold multiple spatial locations on a hexagon in WM whilst answering an unrelated question for each location point shown. The JAJ is similar to the Mr. X task from the Automated Working Memory Assessment (Alloway, Gathercole, Kirkwood, & Elliott, 2008) and earlier versions of similar visuo-spatial tasks (e.g., Shah & Miyake, 1996). Participants are presented with images of two characters, Jack and Jill, both holding a ball in one of their hands. For each image, participants have to (a) decide whether Jack holds the ball in the same hand as Jill and (b) to remember the position of Jack’s ball on a hexagon of dots. At the end of each sequence of images, participants have to indicate the position of the balls in the correct order. The task had 14 trials with the length of the image sequences increasing and hence trials becoming increasingly difficult.

##### Musical working memory tasks

*Rhythm ability test (RAT)*: The RAT (Müllensiefen, Fiedler, Andrade, Forth, & Frieler, 2020) measures memory for non-pitched rhythmic stimuli and is related to the musical sequence transcription task described by Zuk, Andrade, Andrade, Gardiner, & Gaab (2013a, b). Each trial of the RAT comprises the playback a rhythmic pattern of high-frequency claps and low-frequency bass drum kicks. After the pattern is played, visual representations of four different rhythms are shown with light blue squares which representing claps and dark blue squares which representing the bass drum kick. Participants are required to click on the visual representation which corresponds to the rhythmic pattern they have just heard. The RAT comprized 16 trials of increasing difficulty as a function of number of rhythmic events, the complexity of the rhythmic sequence, and the similarity of the target sequence to the three lures.

*Melodic discrimination test (MDT)*: Melodic discrimination ability was assessed using the adaptive melodic discrimination test (MDT; Harrison et al., 2017). This test uses a 3-AFC response task with each item consisting of three versions of a melody at different transpositions in pitch. Two of these versions are always identical and one is always different. The participant’s task is to identify the nonidentical melody (the ‘odd-one out’), but to ignore transpositions between versions. The version of the MDT used in this study comprized 20-item items using the adaptive procedure as described in the original study (Harrison et al., 2017).

##### Psychometric questionnaires

As per Study 1, two questionnaires were also administered. The Gold-MSI (Müllensiefen et al., 2014) assessed general musical sophistication, as well as different aspects of musical expertise and skills on five different subscales (i.e., active musical engagement, perceptual abilities, musical training, singing abilities, and emotional use of music). The BAIS (Halpern, 2015) measured auditory imagery ability via two separate subscales: vividness of auditory imagery and auditory imagery control. While some self-report items of the BAIS ask to imagine musical contents (e.g., the voice of an opera singer; the sound of a rock song on the radio), others make reference to non-musical auditory elements (e.g., the sound of gentle rain, the cheer of the crowd at a sports game). In this way, the BAIS is not measuring an exclusively musical imagery ability, but rather general auditory imagery ability.

#### Procedure

Participants completed all tasks in computer testing booths, so that compliance to the tasks could be monitored. All tasks were introduced with written instructions, and practice trials were provided with feedback. During each of the tasks, no feedback was given. On the first visit, all participants completed the 6 tasks in the following order using a common online user interface: Backward Digit Span (BDS), Memory Updating Figural (MUF), Jack and Jill (JAJ) visuo-spatial WM test, Rhythm Memory Test (RAT), aPIAT, and Melodic Discrimination Test (MDT). They then completed the Gold-MSI and BAIS self-report questionnaires. Testing for timepoint 1 took approximately 1 h. Between 7 and 14 days later, participants returned and completed the RAT and PIAT again. Testing for timepoint 2 took approximately 20 min. All participants were invited to return; however, only *n* = 66 (46%) completed the task at both timepoints. Due to computer error, some participants’ scores for some tests and questionnaires were lost, including 2 participants who did not complete the aPIAT.

Validity of the aPIAT was obtained through correlations with other WM Tasks, as well as with psychometric questionnaires. Reliability of the aPIAT was assessed through two separate measures: test–retest reliability and IRT standard error. Test–retest reliability describes the consistency of test scores over repeated testing sessions; it is measured here as the Pearson correlation between ability estimates measured at timepoints 1 and 2. Unlike test–retest reliability, IRT standard errors have the advantage that they can be computed from a single test session. However, they do rely on the assumptions of the underlying IRT model. The validity and reliability were also calculated across varying test item lengths of the aPIAT.

#### Ethics

The experiment received ethical approval by the Ethics committee at both Goldsmiths, University of London and Macquarie University.

### Results

Validity was obtained through calculating correlations between aPIAT scores and the other WM tasks, as well as psychometric questionnaires. Correlations of aPIAT scores with all three non-musical WM measures show the expected pattern of moderate correlations (0.44 ≥ *r* ≥ 0.42) and stronger correlations (0.57 ≥ *r* ≥ 0.54) which were seen with the two musical WM tests (Table 5). The number of participants included in the correlation calculations are as indicated. Correlations with aspects of self-report musical sophistication are equally strong, especially with self-reported musical training (0.57), perceptual abilities (0.44), singing abilities (0.45), and general musical sophistication (0.53). Correlations with self-reported auditory imagery ability are somewhat lower (0.24 for vividness and 0.30 for auditory imagery control) (see Table 6).

**Table 5:** Correlations and 95% confidence intervals of aPIAT scores with other measures of non-musical and musical working memory (WM)

	Non-musical WM			Musical WM	
	Backward digit span (BDS)	Jack and Jill (JAJ)	Memory updating figural (MUF)	Melodic discrimination (MDT)	Rhythm ability (RAT)
N	143	137	142	143	142
aPIAT Score	0.43*** [0.286, 0.555]	0.44*** [0.294, 0.566]	0.42*** [0.274, 0.547]	0.57*** [0.448, 0.671]	0.54*** [0.412, 0.647]

Significance is denoted as ***p < 0.001 [corrected for multiple comparisons using Holm’s (1979) procedure]

**Table 6:** Correlations and 95% confidence intervals of aPIAT scores with self-reported musical sophistication (Gold-MSI) and auditory imagery ability (BAIS)

	*N*	aPIAT Score	
**Gold-MSI subscales**			
Age	142	0.001	[− 0.164, 0.166]
Gender	143	0.09	[− 0.075, 0.250]
Active engagement	143	0.33**	[.175, 0.469]
Emotions	143	0.24	[0.079, 0.389]
Musical training	143	0.57***	[0.448, 0.671]
Perceptual abilities	143	0.44***	[0.297, 0.563]
Singing abilities	143	0.45***	[0.309, 0.572]
General sophistication	143	0.53***	[0.401, 0.639]
**BAIS subscales**			
Vividness	139	0.24	[0.077, 0.391]
Control	139	0.30*	[0.141, 0.444]

Significance is denoted as ***p < 0.001, **p < 0.01, *p < 0.05 [corrected for multiple comparisons using Holm’s (1979) procedure]

The test–retest reliability for the 25-item version of the aPIAT was *r* (64) = 0.65 (95% CI: [0.48, 0.77], *p* < 0.001) and this IRT version has a mean standard error of measurement (as computed from the of the first test session) of 0.74 (median = 0.61).

**Fig. 3:**
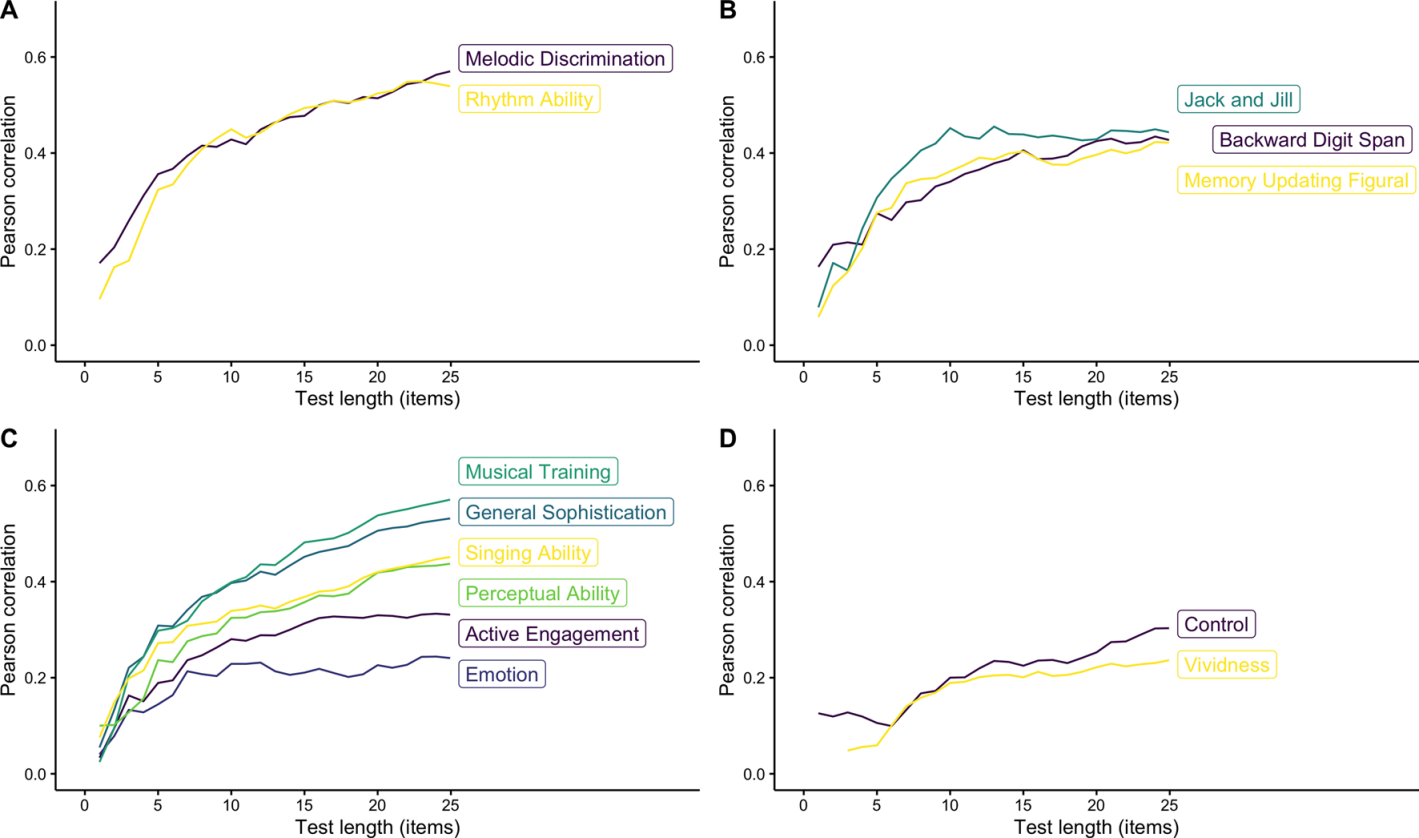
Pearson correlations between aPIAT scores and related measures as a function of aPIAT test length. **a** Musical WM tests. **b** Non-musical WM tests. **c** Subscales of the Goldsmiths Musical Sophistication Index (Gold-MSI) questionnaire. **d** Subscales of the Bucknell Auditory Imagery Scale (BAIS)

In addition to correlations with final aPIAT scores calculated above, Fig. 3 shows how the correlations between aPIAT scores and other WM scores, as well as the self-reported musical sophistication and auditory imagery abilities change as the number of trials of the aPIAT increases. While correlations with non-musical measures of WM plateau after about 15 items (Fig. 3b), correlations with musical measures of WM continue increasing as trials are added up to the maximum of 25 (Fig. 3a). Similarly, most of the aPIAT score correlations with self-report measures of musical sophistication (Fig. 3c) and auditory imagery abilities (Fig. 3d) gradually increase with more trials. Put together, these results suggest that the validity of the aPIAT gradually increases with more trials, with no evidence of a ceiling effect within the range considered (1–25 items).

**Fig. 4:**
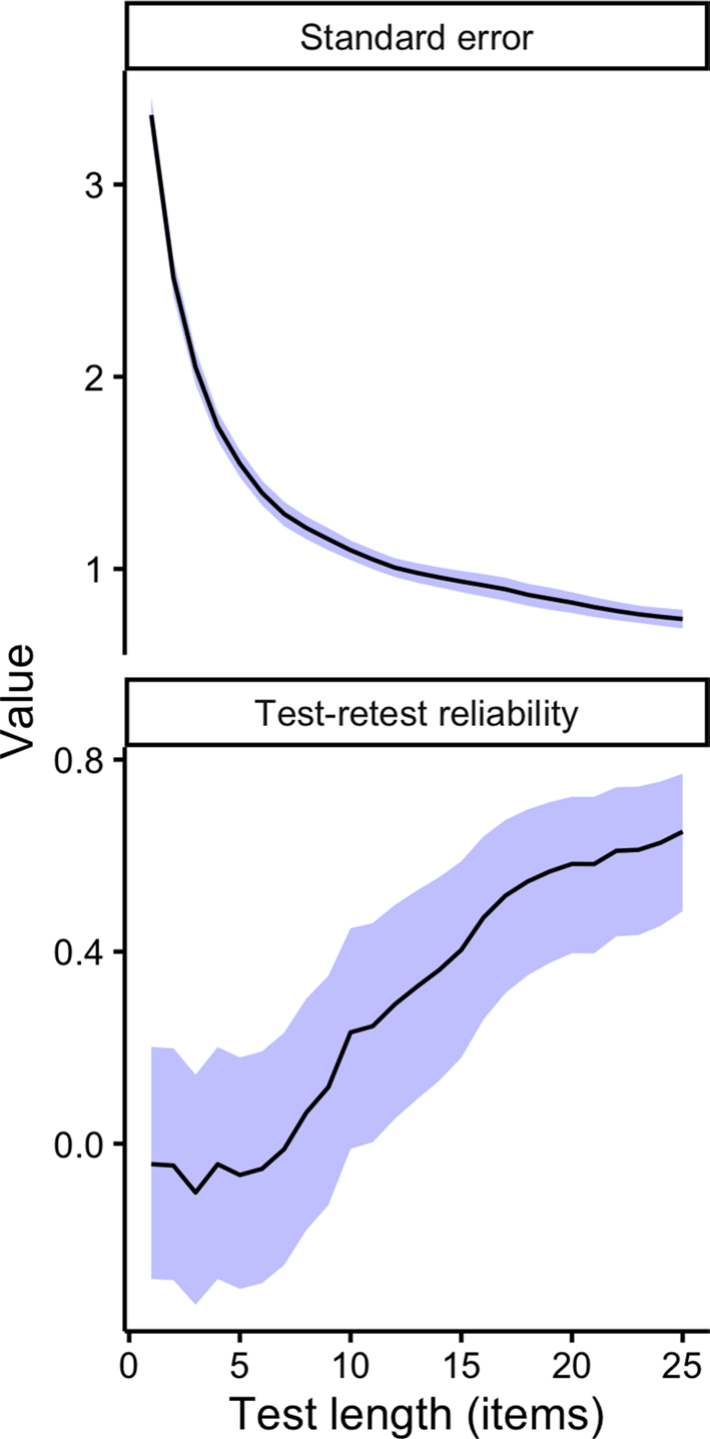
Reliability metrics for the aPIAT as a function of test length. ‘Standard error’ corresponds to the (mean) standard error of aPIAT ability estimates at timepoint 1, as computed by the IRT model (144 participants). ‘Test–retest reliability’ corresponds to the Pearson correlation coefficient between aPIAT ability estimates at timepoints 1 and 2 (66 participants). The shaded regions plot 95% confidence intervals

The reliability measures of test–retest correlation and IRT standard error of measurement were also plotted as a function of test length of the aPIAT in Fig. 4. As expected, reliability increased with longer test lengths, with test–retest reliability growing from 0.23 (10 items) to 0.65 (25 items) and mean standard error shrinking from 1.10 (10 items) to 0.74 (25 items).

### Discussion

The aim of Study 3 was to test the validity and reliability of the aPIAT against a number of other working memory measures. As predicted, the strongest correlations were found between musical working memory tasks and aPIAT. However moderate correlations were also seen between the aPIAT and non-musical working memory tasks. Although the test–retest reliability of the 25-item aPIAT was only 0.67, this is similar to other musical 2AFC tasks having a comparable number of items (Harrison & Müllensiefen, 2018; Larrouy-Maestri, Harrison, & Müllensiefen, 2019; Law & Zentner, 2012).

In addition, analysis of validity and reliability over varying item lengths of the aPIAT revealed that the validity of the aPIAT gradually increases with more trials, with no evidence of a ceiling effect within the range considered (1–25 items). Test–retest correlations were particularly low for very short tests (length less than 10 items). This suggests that early test responses could be less informative than later test responses, perhaps because participant performance only becomes reliable after accumulating sufficient practice. Future work could address this by expanding the test’s training phase, or by omitting early items from ability scoring.

In sum, these analyses of validity and reliability as a function of test length indicate that, as expected, shortening the adaptive PIAT below 25 items negatively impacts validity and reliability. There is no clear evidence for a ceiling effect for either validity or reliability, and so, we advise using the full 25 items where practical. Nonetheless, in situations where time is limited and shortening is imperative, the researcher can use Figs. 3 and 4 to find a principled balance of reliability, validity, and test length.

Finally, as per the results of Study 1 and 2, performance on the aPIAT was not correlated with age or gender, and showed a greater correlation with the BAIS-C than for BAIS-V. This is to be expected, since the aPIAT requires both manipulation and maintenance of musical images, and the BAIS-C subscale measures the self-report ability to mentally change one sound image to another. Consistent with Study 1 and 2, performance on the aPIAT also strongly positively correlated with the musical training subscale of the Gold-MSI, suggesting participants with greater musical training show enhanced imagery ability, consistent with previous research (Aleman et al., 2000). The results are also consistent with a recent study using a modified PIAT task that showed pitch imagery performance partially mediated the relationship between pitch short-term memory and pitch imitation (singing) ability (Greenspon & Pfordresher, 2019).

## General discussion

The PIAT was designed to address many of the shortcomings of other tasks used to measure pitch imagery ability. However, the original PIAT was time-consuming to administer (approx. 1 h) and experienced as tedious by participants. This series of studies sought to develop a more efficient test of auditory mental imagery ability through psychometric development of the PIAT in three phases—exploration, calibration, and validation. The outcome of these studies is a reliable 25-item validated test that can be administered locally online within 8 min and provides a single aPIAT score of pitch imagery ability. The modelling process of the PIAT/aPIAT also provided new insights into auditory imagery itself.

The explanatory item response model of the aPIAT features two variables that suggest perceptual biases in the task: the probability of the probe given the last heard note and the range of notes heard in the set-up component. Cognitive or perceptual biases in music perception are rarely studied systematically. While studies have shown visuo-spatial biases in pitch perception (Connell, Cai, & Holler, 2013) and perceptual biases in time perception towards regular rhythmic grouping and intensity (Penel & Drake, 2004), the role of perceptual bias is often difficult to detangle from the requirements of a given task. Hence, the aPIAT, which is based on an explanatory item response model that uses features of individual items as predictors, provides a good opportunity to demonstrate and quantify the effect of these perceptual biases in future applications of the task.

The model contributes to our understanding of Edwin Gordon’s concept of ‘audiation’ (Gordon, 1985, 1989b, 1999), specifically to the subtype of audiation that requires hearing music, in particular pitch, in the mind. Gordon’s test batteries mainly rely on a simple same–different paradigm (i.e., hear a melody, insert pause, hear another melody, and have participants indicate if the second melody was the same or different compared to the first one) where patterns differed either in pitch or rhythm (Gordon, 1989a). While Gordon does not provide a cognitive model of the processes underlying the performance on his tests, recently cognitive models of melodic discrimination tests have pointed to memory and similarity comparison as two core components (Harrison et al., 2016). However, these same–difference tests cannot be simply equated to musical aptitude or melodic memory abilities as they draw on a number of distinct cognitive processes which contribute to individual differences (Harrison et al., 2016). In addition, Gordon’s tests do not require the internal mental manipulation of sounds or musical elements, which is the core component of his audition concept (Gordon, 1989b). In contrast, the aPIAT explicitly requires internal manipulation (as well as memory and similarity comparison) as part of the cognitive process for solving the task, making it a more suitable test for assessing auditory imagery ability and audiation skills as formulated in Gordon’s theory. The results of the current studies show a positive association between self-reported musical training as well as perceptual abilities, and ability on the aPIAT. Hence, future work will use the aPIAT longitudinally to assess auditory imagery ability as children develop their musical skills, and to determine whether this ability to maintain and manipulate tones can in fact serve as a predictor of musical aptitude as well as non-musical development. The correlations with core indicators of skilled musical expertise and cognitive capacity are very encouraging in this perspective.

In conclusion, ability on the aPIAT requires the skill to both maintain and manipulate tones in mental imagery, as well as to resist perceptual biases that can lead to incorrect responses. The current validation study has demonstrated substantial correlations of the aPIAT with established measures of musical and non-musical working memory as well as with self-reported musical expertise and skills.

More broadly, the aPIAT can be used as a short and efficient test of a core musical ability and combined with other musical and cognitive tasks (Gordon, 1989a; Law & Zentner, 2012; Ullén, Mosing, Holm, Eriksson, & Madison, 2014; Wallentin, Nielsen, Friis-Olivarius, Vuust, & Vuust, 2010) as part of larger batteries. It is an ideal tool in which to address questions of auditory imagery ability and musicality. The test is freely available and suitable either for laboratory testing or online testing.^1^

## Appendix 1

See Table

**Table 7:** Predictor variable names, definitions, and range of values

Predictor Variable	Definition	Range of values	Study 1	Study 2	Study 3
Level	Number of imagined tones (i.e., silent arrows) per trial	1:5	✔	✔	1:6
Heard_Range	The number of unique tones played during the set-up period, including the start note	2:5	✔	✔	✔
ProbabilityProbe_LastHeard	Probability of probe based on actual data of distance between last heard note and probe	0.0003:0.40	✔	✔	✔
ProbeTrueIm_AbsDiff	Absolute difference between the true imagined final note and the probe presented	0, 1, 2	✔	✔	✔
Stage 1	Takes a value of 1 when trials where start note is tonic, number of arrows in set-up sequence is 3 and Key is C Major	0/1	✔		
Stage 2	Takes a value of 1 when trials where start note is tonic, and number arrows in set-up sequence is 3–5	0/1	✔		
Stage 3	Takes a value of 1 when trials where start note is dominant, and number arrows in set-up sequence is 3–6	0/1	✔		
Stage 4	Takes a value of 1 when trials where start note is tonic or dominant, and number arrows in set-up sequence is 3–6. Stage 4 trials only reached when participants successfully completed Level 5—Stage 3	0/1	✔		
ProbeNote_Is_StartNote	Takes value of 1 if probe was start note, and 0 if it was not	0/1	✔		
Probability_Probe	Probability of the probe, given the total number of arrows presented in the trial	0:0.375	✔		
Key	Key signature (1–5 corresponding to C Maj, C# Maj, D Maj, Eb Maj, E Maj)	1:5	✔	✔	
Start_Note	Tonic (1) or Dominant (0) of scale	1/0	✔	✔	
Heard_Arrow	Number of sounded arrows presented in set-up period	3:5	✔	✔	
Probe_Start Note_ Difference	Probe number relative to steps away from the start note (at 0)	− 4: + 4	✔	✔	
Probe	This is the probe note number in the scale where 1 is the tonic up to 8 which is the tonic up one octave, − 4, − 5, − 6, and − 7 are the 4th, 5th, 6th, and 7th notes of the scale in the lower octave	− 4:− 7; 1:9	✔	✔	
Probe_Previous	Whether the probe was previously heard in the initial set-up period of the trial (1) or not (0)	1/0	✔	✔	
Low_Probe	If probe was not heard in set-up and was lower than start note (so not heard in initial scale), then (1) else (0)	1/0	✔	✔	
LastHeard	The last note heard in the sequence relative to start note	− 3: 3	✔	✔	
Binomial_ Probability_Probe_ StartNote	Binomial probability of probe based on start note	.003:.21	✔	✔	
Probability_Probe_ StartNote	Probability of probe based on actual data of distance between start note and probe	0.15:0.31	✔	✔	
Binomial_ Probability_Probe_ LastHeard	Binomial probability of probe based on last heard note	0.009:0.24	✔	✔	
ProbeLastHeard_ AbsDiff	Absolute value of the difference between last heard note and probe	0:6	✔	✔	
ProbeNote_1	Takes a value of 1 only if the probe is the tonic; is derived from Probe = 1 or 8	1/0	✔	✔	
ProbeNote_1or5	Takes a value of 1 only if the probe is the tonic or dominant; i.e., Probe = − 5, 1, 5, or 8	1/0	✔	✔	
Direction_Same	Takes value of 1 only if the direction of the probe tone from the last note heard (up, down, same) is the same as the direction of the true imagine tone from the last note heard	1/0	✔	✔	
LastHeardTrueIm_AbsDiff	Absolute value of the difference between last heard note and true imagined final note	0:4	✔	✔	
TrueIm _1or5	Takes a value of 1 only if the true imagined final note is the tonic or dominant; is derived from true imagined note = − 5, 1, 5 or 8	1/0	✔	✔	
ProbabilityProbe_Constrained	Probability of the probe, given the total number of arrows presented in the trial and the restraint that the start note cannot be used as a correct probe	0:0.4		✔	

Study number column indicates with a tick when the variable was used in modelling for that Study

7.

## Footnotes

1 The test is available at the open-source repository https://github.com/pmcharrison/piat and the permanent archive https://doi.org/10.5281/zenodo.3239098.
